# **Pentagalloyl glucose inhibits TNF**‐**α**‐**activated CXCL1/GRO-α expression and induces apoptosis**‐**related genes in triple-negative breast cancer cells**

**DOI:** 10.1038/s41598-021-85090-z

**Published:** 2021-03-11

**Authors:** Patricia Mendonca, Sumaih Alghamdi, Samia Messeha, Karam F. A. Soliman

**Affiliations:** Division of Pharmaceutical Sciences, College of Pharmacy and Pharmaceutical Sciences, Institute of Public Health, Florida A&M University, Room G134 H Pharmacy Building, 1415 ML King Blvd, Tallahassee, FL 32307 USA

**Keywords:** Receptor pharmacology, Breast cancer

## Abstract

In triple-negative breast cancer (TNBC), the tumor microenvironment is associated with increased proliferation, suppressing apoptotic mechanisms, an altered immune response, and drug resistance. The current investigation was designed to examine the natural compound pentagalloyl glucose (PGG) effects on TNF-α activated TNBC cell lines, MDA-MB-231 and MDA-MB-468. The results obtained showed that PGG reduced the expression of the cytokine GRO-α/CXCL1. PGG also inhibited *IƙBKE* and *MAPK1* genes and the protein expression of IƙBKE and MAPK, indicating that GRO-α downregulation is possibly through NFƙB and MAPK signaling pathway. PGG also inhibited cell proliferation in both cell lines. Moreover, PGG induced apoptosis, modulating caspases, and TNF superfamily receptor genes. It also augmented mRNA of receptors *DR4* and *DR5* expression, which binds to TNF-related apoptosis-induced ligand, a potent and specific stimulator of apoptosis in tumors. Remarkably, PGG induced a 154-fold increase in *TNF* expression in MDA-MB-468 compared to a 14.6-fold increase in MDA-MB-231 cells. These findings indicate PGG anti-cancer ability in inhibiting tumor cell proliferation and GRO-α release and inducing apoptosis by increasing *TNF* and TNF family receptors' expression. Thus, PGG use may be recommended as an adjunct therapy for TNBC to increase chemotherapy effectiveness and prevent cancer progression.

## Introduction

In the US, breast cancer (BC) is the second leading cause of mortality among women. In breast cancer treatment, one of the most significant challenges in treating this disease is chemotherapeutic resistance. However, it is well-known that chemotherapeutic resistance in BC is associated with abnormal growth factor signaling and aberrant hormonal response. The exact mechanisms of this phenomenon in cancer treatment are still unknown^1,2^. Current insights in molecular targets lead to the recognition of BC therapy in the subset HER2/neu. Regardless of new advances, no treatments for triple-negative breast cancers (TNBC) have been identified. TNBC patients, which present a lack of estrogen receptor (ER), progesterone receptor (PR), and basal level of the HER2/neu oncogene, have an aggressive phenotype, poor prognosis, with an increased chance of metastasis and deficiency of specific and therapeutic targets^1,3–6^. The tumor microenvironment (TME), observed in TNBC, is linked to increased cell proliferation, migration, angiogenesis, suppression of apoptotic mechanisms, an altered immune response, and drug resistance. The oncogenic transformations in the TME involve the infiltration of immune cells and the stimulated fibroblasts that produce cytokines, chemokines, and growth factors, stimulating tumor initiation and progression^7,8^. Chemokines and cytokines may affect tumor immunity and cancer progression directly or indirectly, impacting cancer therapy outcomes^9^. Meanwhile, recent investigations linked TME to tumor heterogeneity, apoptosis repression, oncogenic genes overexpression, activation of tumor suppressors, and deregulation of signal transduction pathways^10–13^. On the other hand, more studies have focused on finding molecules and pathways that can successfully induce apoptosis and tumor regression, impeding cancer cell progression without affecting normal cells^14^.

The current investigation aims to examine the modulating effect of the polyphenolic natural compound pentagalloyl glucose (PGG), found in many medicinal herbs, on cytokine release. In vivo and in vitro investigations indicated the potential use of PGG in the treatment and prevention of many cancers and inflammatory diseases^15–17^. Our previous studies verified PGG potential in down-regulating pro-inflammatory cytokines in BV-2 microglial cells activated by LPS/IFNγ. In this work, PGG decreased the expression of MCP5 and MMP-9 cytokines, whose overexpression is involved in chronic inflammation and neurodegeneration. Also, MCP-5 and MMP-9 are known to modulate genes and proteins that participate in NFƙB and MAPK signaling, suggesting a possible molecular mechanism of action for the PGG inhibitory effect^18,19^. NFƙB and MAPK signaling regulate several genes associated with inflammatory processes and cancer, controlling cell proliferation and survival, chronic inflammation, apoptosis, and the conversion to cancer cells^20^.

Considering the cytokines' critical function in cancer cell proliferation, metastasis, and angiogenesis, this study hypothesized that PGG would inhibit cytokines' expression, leading to reduced cell proliferation and induction of apoptosis through the modulation of the expression of apoptotic-associated genes. Since the genetic variability of TNBCs contributes significantly to the tumor's microenvironment, the current study evaluated PGG effects on two distinct genetically different TNBC cell lines, MDA-MB-231 (MM-231) and MDA-MB-468 (MM-468) cells.

## Results

### PGG inhibits cell viability and cell growth in MM-231 and MM-468 TNBC cells

**Figure 1:**
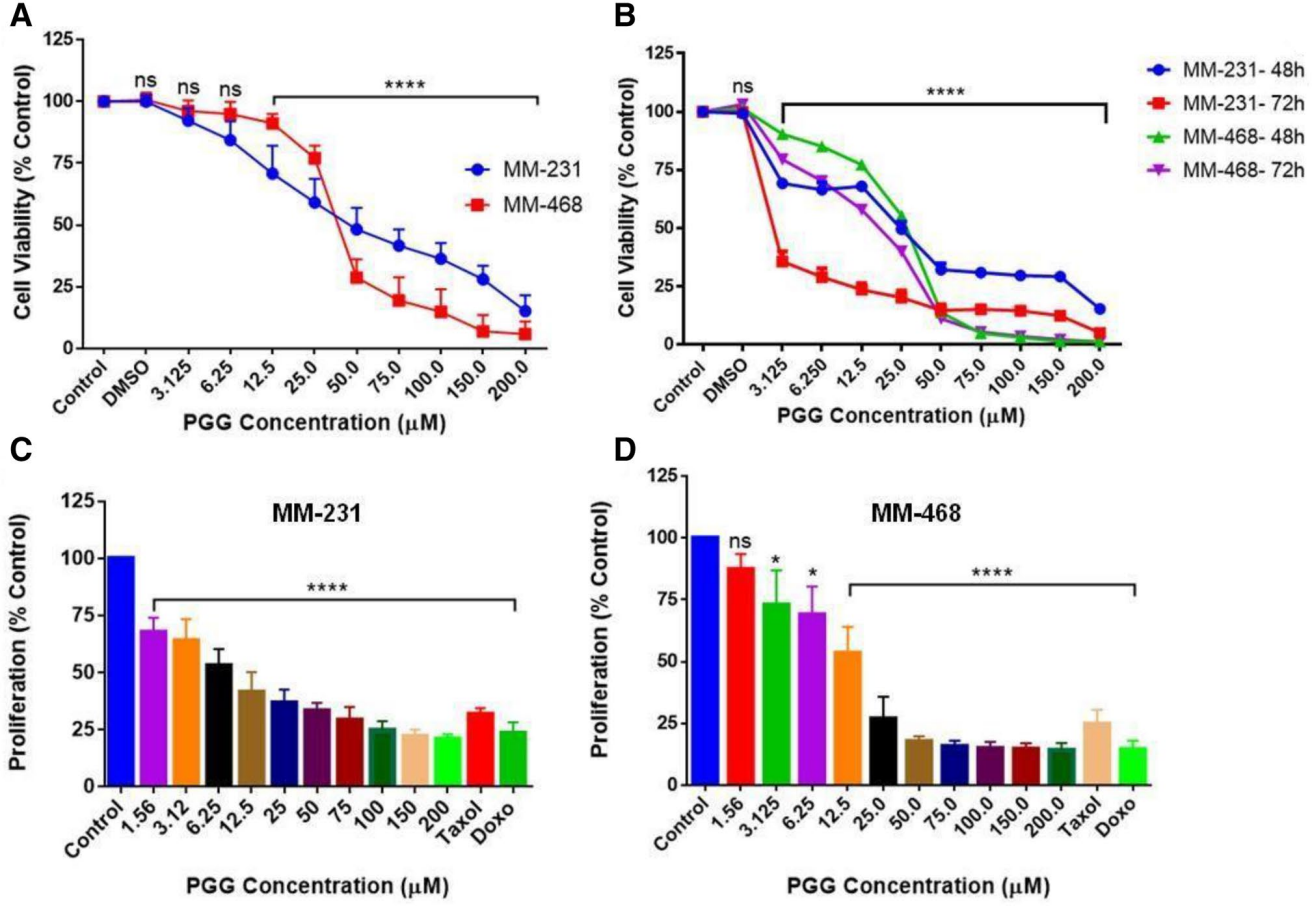
PGG inhibitory effect on cell viability and cell growth in MM-231 and MM-468 TNBC cells. In cell viability studies, PGG concentrations of 3.125–200 µM were tested for 24 (**A**), 48, and 72-h (**B**) treatment periods. In proliferation assays, PGG concentrations of 1.56–200 µM were used. Taxol and doxorubicin (1 µM) were used as positive controls, and treated cells were maintained at 5% CO_2_ and 37 °C for a 96-h incubation period (**C** and **D**). Data represent the mean ± SEM of 3 biological studies (n = 5). Differences between groups (control vs. treatments) were evaluated for statistical significance using a one-way ANOVA and Dunnett's multiple comparison tests. *p < 0.05, ****p < 0.0001, ns = *p* > 0.05.

Cells were treated with PGG (3.125–200 μM) for 24, 48, and 72 h to examine the PGG effect on cell viability. Figure 1A,B show that PGG acted on both cell lines in a dose and time-dependent manner. After 24 h-treatment, PGG concentrations of 12.5 and 25 μM were more effective in MM-231 cells than MM-468. However, in the concentration of 50 μM and over, PGG cytotoxic effect was higher in MM-468 cells (Fig. 1A), showing that PGG affects both cell lines differently. In both cell lines, concentrations lower than 12.5 μM caused a non-significant effect. The IC_50_ of 50.23 ± 2.16 µM for MM-231 and 35.72 ± 0.75 µM for MM-468 indicate that PGG affects the cell lines differently, inducing more significant cytotoxicity in MM-468 cells (Fig. 1A). After 48 and 72 h, the PGG effect was more pronounced compared to the period of 24 h, leading to higher toxicity (Fig. 1B). However, there was higher toxicity in the MM-468 cells in concentrations over 50 μM, following the same pattern observed after the 24 h-period incubation.

To investigate the PGG effect on cell proliferation, TNBC cells were treated with PGG (1.56–200 μM) for 96 h. The results demonstrated that the proliferation was inhibited by PGG in both breast cancer cell lines in a dose-dependent manner (Fig. 1C,D). Interestingly, at lower concentrations of PGG showed to be more potent in MM-231 cells; however, in the concentration of 50 μM, PGG was more effective in MM-468 cells, causing 83% suppression in cell proliferation compared to 67% in MM-231. The same pattern was consistent in concentrations above 50 µM. (Fig. 1C,D).

### PGG inhibits the expression of human pro-inflammatory cytokines GRO and GRO-α/CXCL1 in MM-231 and MM-468 TNBC cells

**Figure 2:**
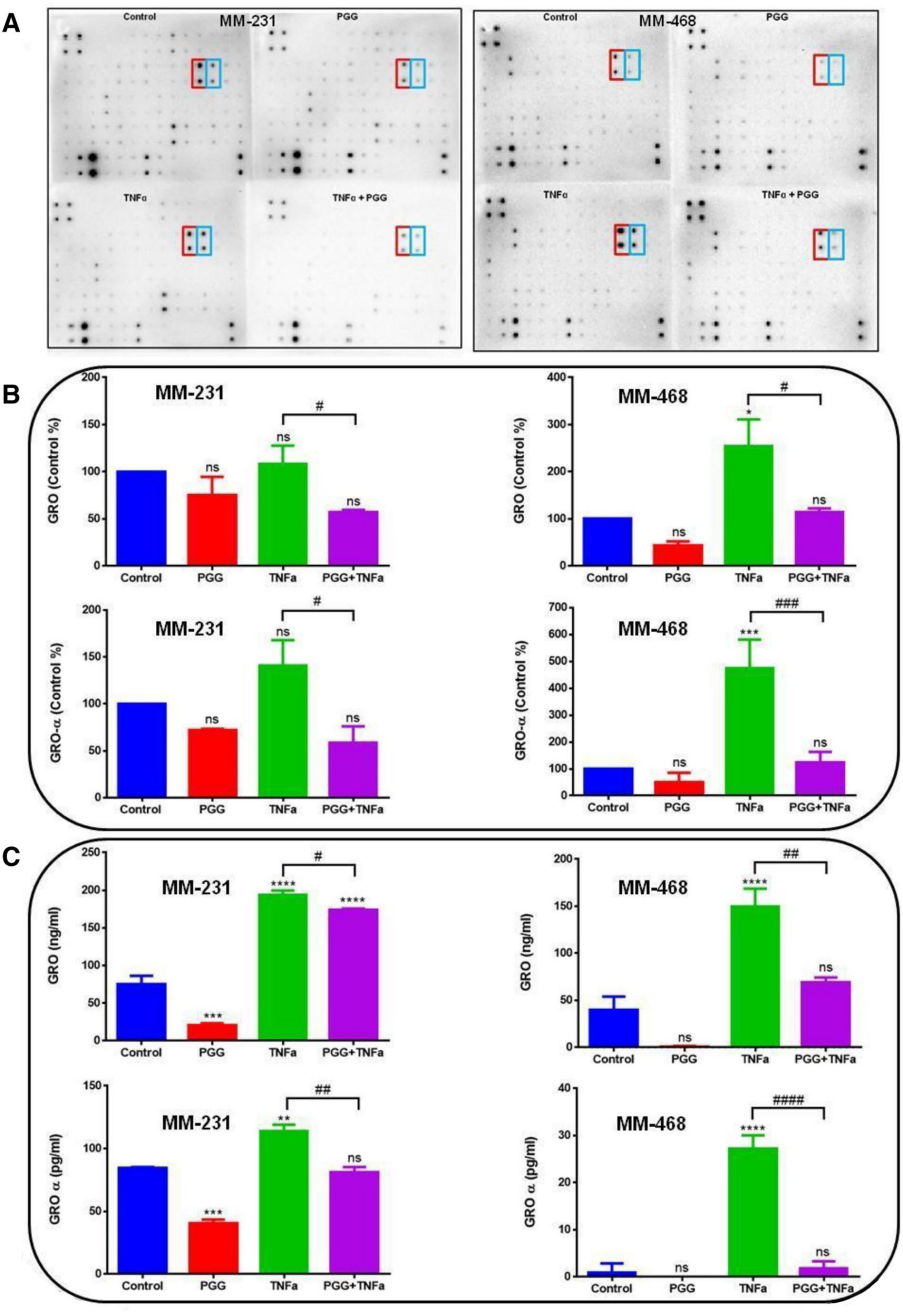
PGG inhibitory effect on the expression of TNF-α-stimulated GRO and GRO-α/CXCL1 in MM-231 and MM-468 TNBC cells using cytokine arrays and ELISA assay. (**A**) Arrays are showing chemiluminescent spot intensity obtained from MM-231 and MM-468 cells supernatants after treatments, presenting the change in protein expression. The red box corresponds to GRO and blue box to GRO-α expression. (**B**) PGG effect on GRO and GRO-α normalized protein expression. Normalized intensities are expressed as control arrays percentages. (**C**) PGG effect on GRO and GRO-α cytokine expression using ELISA quantitative assays. Treatments correspond to control (cells + DMSO), PGG (6.25 and 25 µM for MM-231 and MM-468 cells, respectively), TNF-α (50 ng/ml), and PGG + TNF-α. Mean ± SEM n = 3. Differences between control vs. treatments (*) and TNF-α vs. PGG + TNF-α (^#^) were evaluated for statistical significance by using a one-way ANOVA and Dunnett's multiple comparison tests. *p < 0.05, **p < 0.01, ***p < 0.001, ****p < 0.0001, ^#^*p* < 0.05, ^##^*p* < 0.01, ^###^*p* < 0.001, ^####^*p* < 0.0001, ns = *p* > 0.05.

To investigate and compare the inhibitory effect of PGG on the release of pro-inflammatory cytokines in MM-231 and MM-468 cells, human antibody arrays were used in cytokine arrays and ELISA assay. The results demonstrated that TNF-α upregulated two specific cytokines: growth-related oncogene (GRO) and growth-related oncogene alpha (Gro-α) (Fig. 2A) in both cell lines. GRO detects the alpha, beta, and gamma isoforms in the arrays, and GRO-α detects specifically the GRO-α isoform. The arrays' analysis was performed, and each dot spot intensity was normalized, considering the positive controls from each one of the membranes (RayBio analysis software- RayBiotech). The results demonstrate that TNF-α upregulates the expression of GRO and GRO-α in MM-231 and MM-468 cells compared to the control. However, higher expression was observed in the MM-468 cell line. Contrarily, in the TNF-α-stimulated cells treated with PGG, this compound attenuated TNF-α-induced GRO and GRO-α release significantly in both cell lines. PGG's highest inhibitory effect occurred in GRO-α expression in the MM-468 cells with a fourfold decrease (Fig. 2B). ELISA assays specific for GRO and GRO-α were used to confirm the data from the cytokine arrays. The results confirmed the arrays' findings showing that TNF-α upregulates these proteins' expression in both cell lines compared to the control. Corroborating with the findings from cytokine arrays, in the cells treated with TNF-α + PGG, the expression of GRO and GRO-α was decreased in both cell lines. PGG highest effect was observed in MM-468 cells, showing a 16-fold inhibition compared to the treatment with TNF-α alone (Fig. 2C).

### PGG inhibits GRO-α mRNA expression and modulates IқBKE and *MAPK1* expression in MM-231 and MM-468 TNBC cells

**Figure 3:**
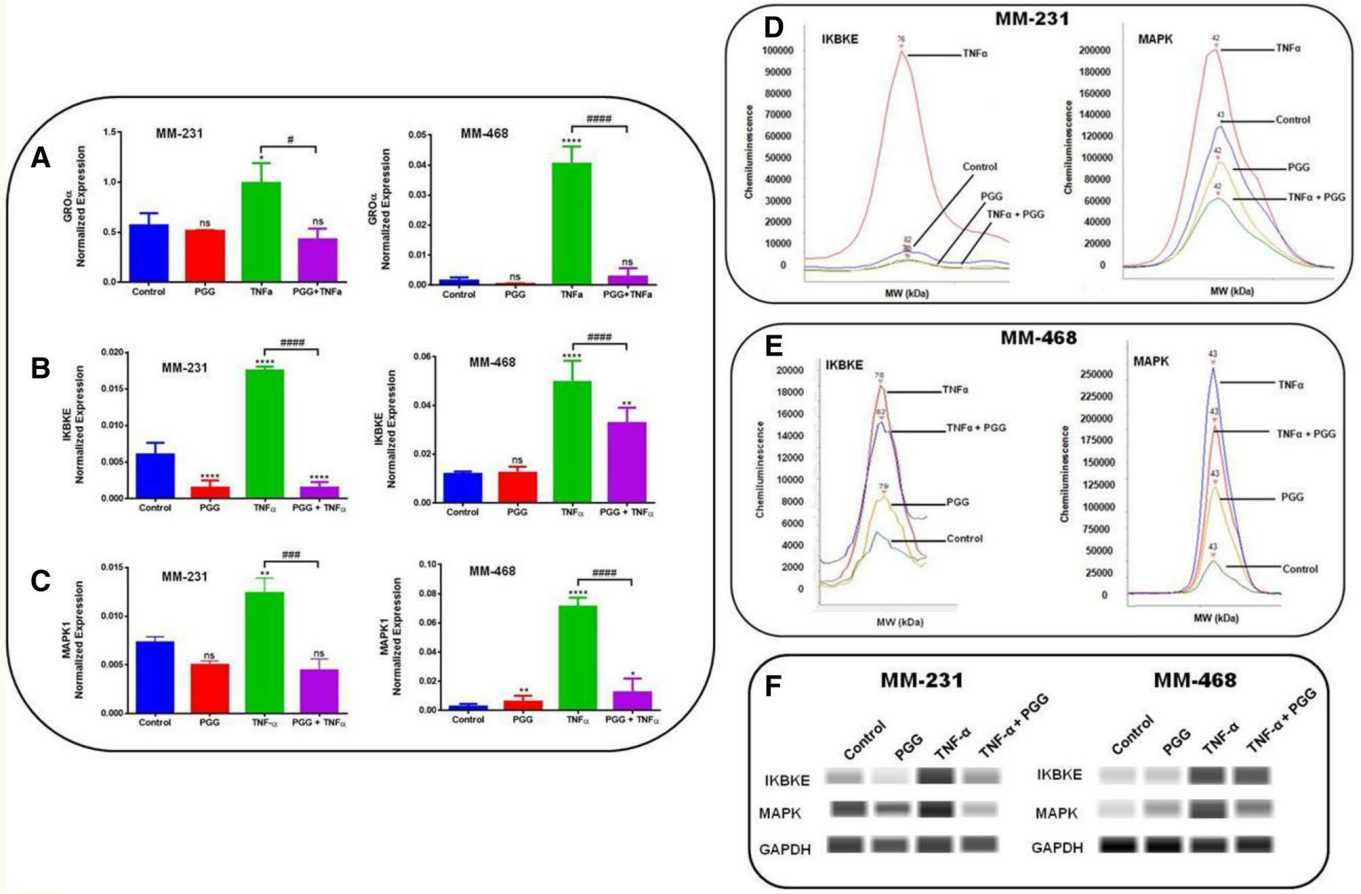
PGG modulatory effect on *GRO-α, IқBKE, and MAPK1* mRNA expression and IқBKE and MAPK protein expression in MM-231 and MM-468 TNBC cells after 24-h treatment. The effect of PGG on *GRO-α*. (**A**), *IқBKE* (**B**), and *MAPK1* (**C**) mRNA expression was investigated in MM-231 and MM-468 TNBC cell lines using RT-PCR. Data refers to the mean ± SEM of three biological experiments (n = 3), corresponding to 4 treatments: control (cells + DMSO), PGG (6.25 and 25 µM for MM-231 and MM-468 TNBC cells, respectively), TNF-α (50 ng/ml), and PGG + TNF-α. Differences between control vs. PGG and TNF-α (*) and TNF-α vs. PGG + TNF-α (^#^) were evaluated for statistical significance by using a one-way ANOVA and Dunnett's multiple comparison tests. *p < 0.05, **p < 0.01, ****p < 0.0001, ^#^*p* < 0.05, ^###^*p* < 0.001, ^####^*p* < 0.001, ns = *p* > 0.05. PGG inhibition of IқBKE and MAPK protein expression was determined through Western analysis. (**D**) and (**E**) show electropherogram representing total IқBKE and MAPK expression after MM-231 and MM-468 cells were exposed to the treatments. (**F**) shows blot view cropped from Compass software corresponding to the protein expression after treatments: Control, PGG, TNF-α, and PGG + TNF-α, respectively. The full-length blots are presented in Supplementary Fig. 1.

To determine the effect of PGG on *GRO-α/CXCL1* mRNA expression in both TNBC cell lines after 24-h treatment, quantitative real-time PCR was performed. TNF-α-induced *GRO-α* expression was statistically significant (*p* < 0.05 (MM-231 cells) (*p* < 0.0001 (MM-468 cells)) in both cell lines, following the same pattern observed in the arrays and ELISA experiments. Comparing TNF-α-treated cells and the ones that received the co-treatment, the results demonstrated that PGG inhibitory effect was higher in MM-468, presenting a 20-fold decrease in the expression of *GRO-α* compared to a twofold in MM-231 cells (Fig. 3A). These data indicate that PGG-associated GRO-α mRNA expression changes follow the same trend detected in the protein studies. To evaluate the possible signaling associated with PGG inhibitory effect over *GRO-α* expression, we explored the PGG effect on mRNA expression of IқBKE and *MAPK1*, which are genes involved in NFƙB and MAPK activation. The data showed that TNF-α upregulated *IқBKE* mRNA expression, with a threefold increase in MM-231 and a fourfold increase in MM-468 cells. TNF-α treatment significantly increased the expression of *MAPK1* mRNA. However, a large increase in the expression was observed in MM-468 cells with a 35-fold contrasted with a twofold increase in MM-231 cells. When TNF-α-stimulated cells were compared to the ones co-treated with TNF-α + PGG, data demonstrated that PGG attenuated both genes' mRNA expression. *IқBKE* mRNA expression presented a downregulation of 91 and 34% and *MAPK1* expression of 64 and 82% in MM-231 and MM-468 cell lines, respectively (Fig. 3B,C). According to these results, PGG treatment is more effective in down-regulating *IқBKE* expression in MM-231 cells; however, it was more efficient in reducing the expression of *MAPK1* in MM-468 cells.

### PGG inhibits IқBKE and MAPK protein expression in MM-231 and MM-468 TNBC cells

The inhibitory effect of PGG on IқBKE and MAPK protein expression after 24-h treatment was investigated using western analysis. The data show that TNF-α increased the expression of both proteins in MM-231 and MM-468 cells. TNF-α-induced IқBKE expression was five times higher in the MM-231 cell line than the MM-468 cell line; however, MAPK expression was higher in MM-468 cells. PGG co-treatment significantly reduced IқBKE and MAPK expression in MM-231, with a higher decrease in IқBKE. PGG also decreased the expression of these proteins in MM-468 cells but with lower levels (Fig. 3D–F). These data demonstrate that IқBKE and MAPK may be involved in NFқB and MAPK signaling pathway and the associated TNF-α-induced GRO-α release and its down-regulation by PGG.

### PGG induces apoptosis and modulates genes associated with the apoptotic process in MM-231 and MM-468 TNBC cells

**Figure 4:**
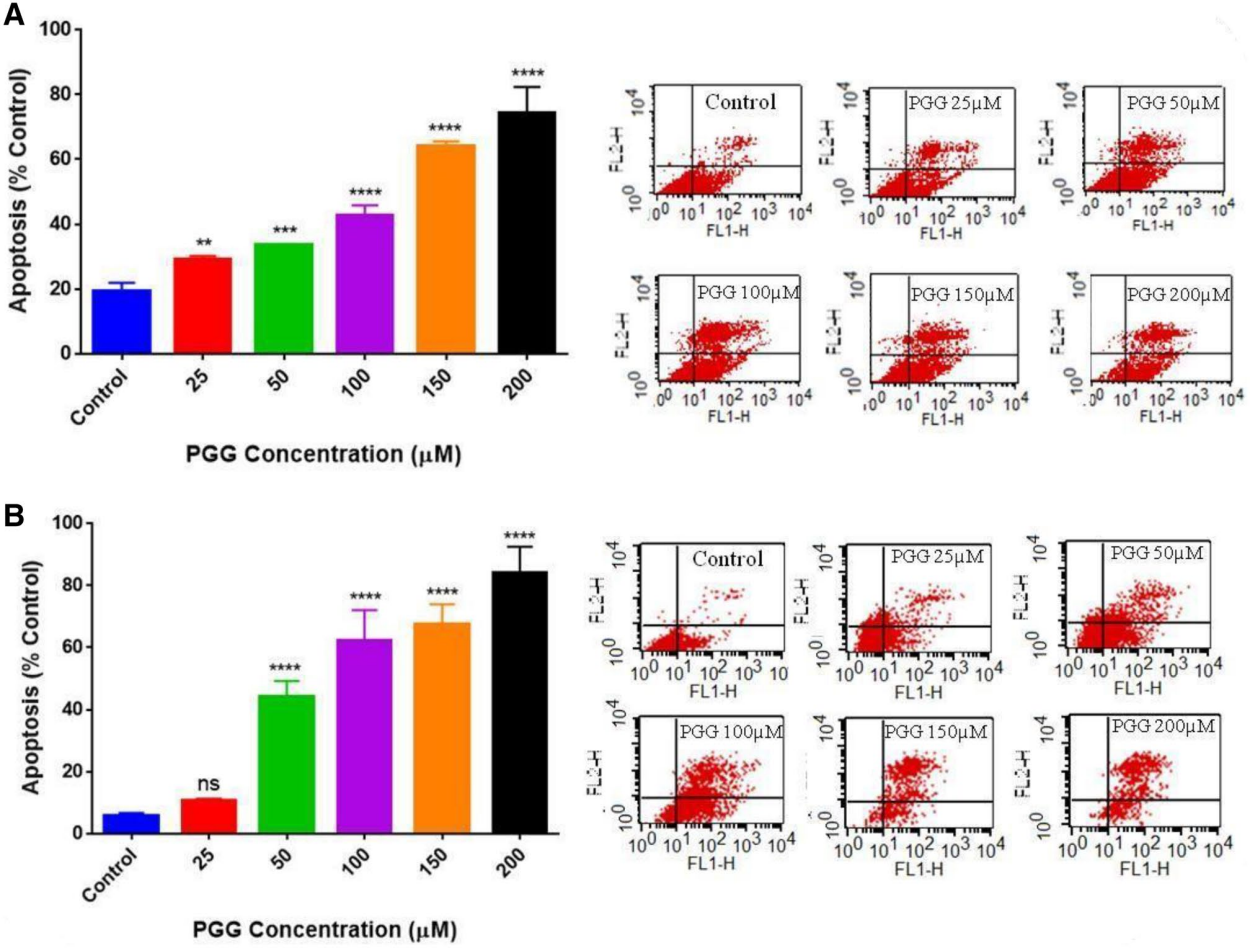
PGG apoptotic effect on (**A**) MM-231 and (**B**) MM-468 TNBC cell lines. Cells were treated with PGG (25–200 µM) and DMSO (< 0.1%) for 24 h. Annexin V-FITC kit was used, and the apoptotic effect was measured using flow cytometry. Results represent the mean ± SEM of 2 biological studies (n = 3). Differences between groups (control vs. treatments) were evaluated for statistical significance using a one-way ANOVA and Dunnett's multiple comparison tests. **p < 0.01, ***p < 0.001, ****p < 0.0001, ns = *p* > 0.05.

**Figure 5:**
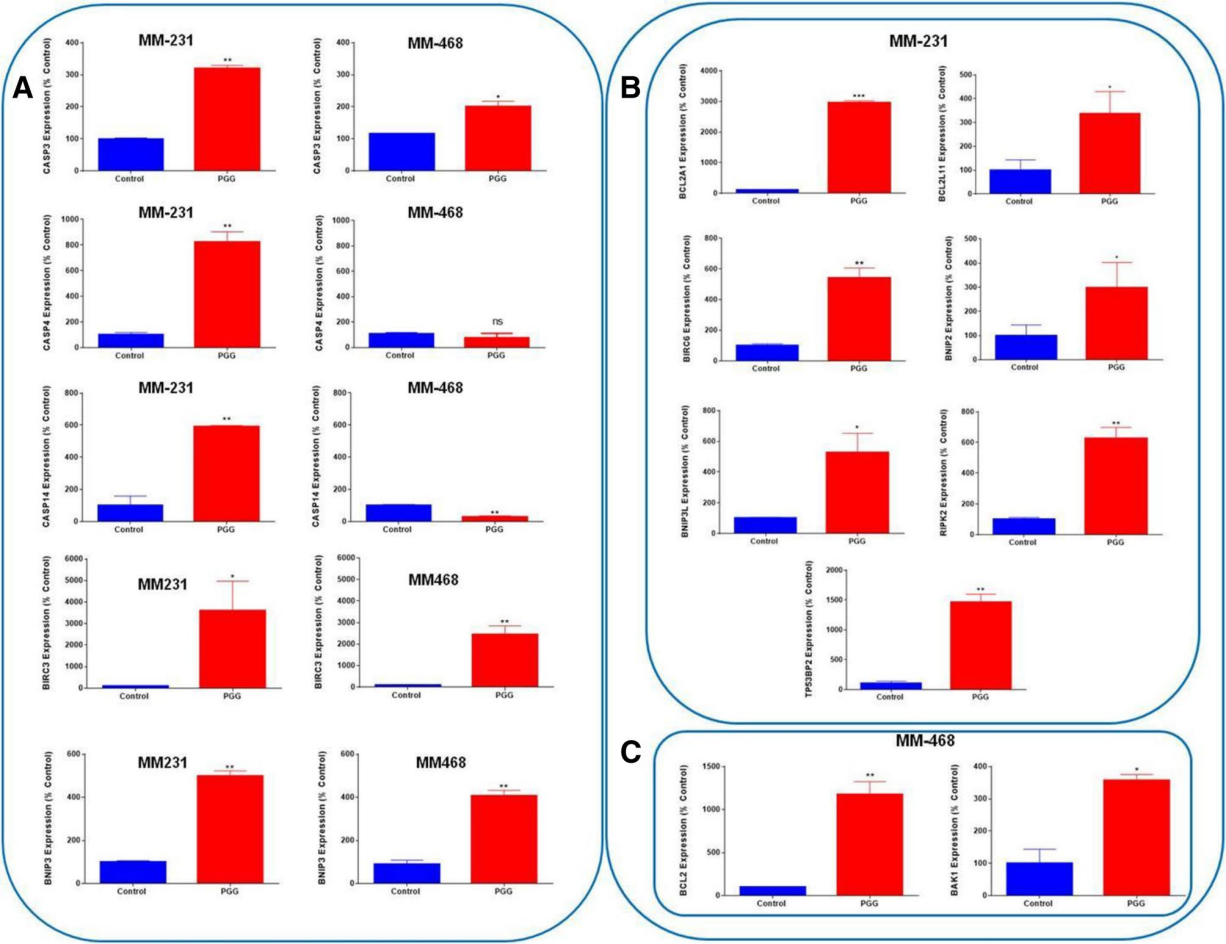
PGG-inducing effect on specific apoptotic gene expression in MM-231 or MM-468 TNBC cell lines using RT-PCR. In (**A**) PGG stimulatory effect on caspases, *BIRC3*, and *BNIP3* gene expression in MM-231 and MM-468 TNBC cell lines. In (**B**) PGG inducing effect on genes specific to MM-231 Cells, and in (**C**) genes specific to MM-468 cells. MM-231 cells were treated with 100 μM and MM-468 cells, with 50 μM of PGG. DMSO (< 0.1%) was used to treat the control cells. Gene expression was calculated based on differences in mRNA expression for each treatment compared to the reference gene (GAPDH). Results represent the mean ± SEM of 3 biological studies (n = 3). Differences between control vs. PGG treatment was evaluated for statistical significance using Student’s T-test *p < 0.05, **p < 0.01, ns =  > 0.05.

**Figure 6:**
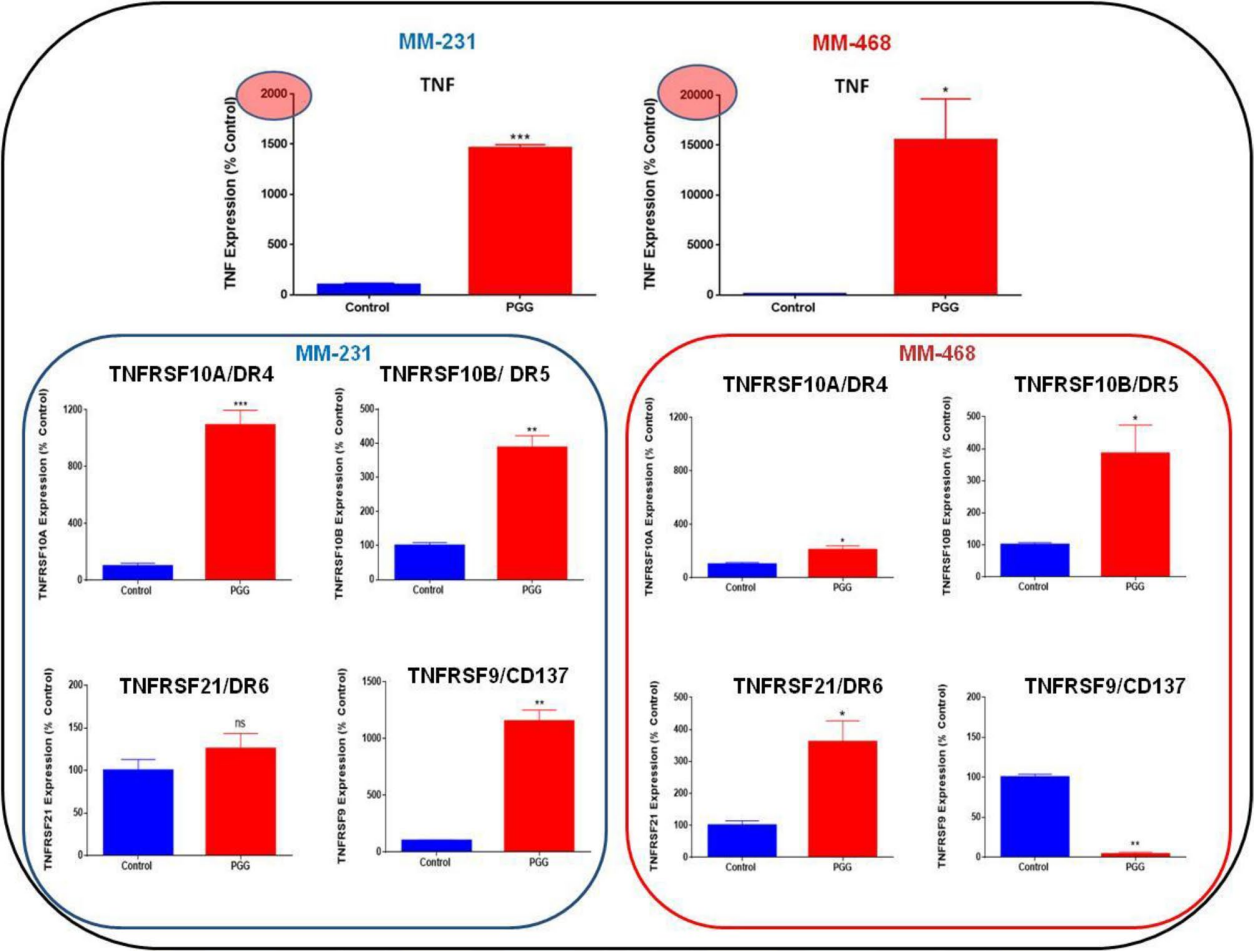
PGG stimulatory effect on TNF and TNF receptors gene expression in MM-231 and MM-468 TNBC cell lines using RT-PCR. MM-231 cells were treated with 100 μM and MM-468 cells, with 50 μM of PGG. DMSO (< 0.1%) was used to treat the control cells. Gene expression was calculated based on differences in mRNA expression for each treatment compared to the reference gene (GAPDH). Results represent the mean ± SEM of 3 biological studies (n = 3). Differences between control vs. PGG treatment was evaluated for statistical significance using Student’s T-test *p < 0.05, **p < 0.01, ***p < 0.001, ns =  > 0.05.

PGG's role in inducing apoptosis was investigated in MM-231 and MM-468 TNBC cells. The treatment consisted of concentrations of PGG, ranging from 25 to 200 μM. PGG apoptotic effect was measured after 24 h using the Annexin V apoptosis kit. PGG induced apoptosis in a dose–response manner in MM-231 and MM-468 (Fig. 4A,B). In MM-231 cells, PGG lower concentration of 25 µM had a significant (*p* < 0.01) apoptotic effect (Fig. 4A). In contrast, MM-468 cells, using the same concentration, did not present a statistically significant difference compared to the control (Fig. 4B). However, in concentrations above 25 μM of PGG, MM-468 cells were more sensitive to the compound's effect, showing a higher percentage of apoptosis compared to the MM-231 cells. PGG's highest concentration (200 μM) induced 74 and 84% of apoptosis in MM-231 and MM-468 cells, respectively (Fig. 4A,B). Based on the flow cytometry findings, a specific concentration of PGG, which induced 40% of apoptosis (100 µM- MM-231 cells and 50 µM- MM-468), was selected. These concentrations were used in RT-PCR apoptosis arrays to investigate PGG influence in the mRNA expression of 89 different signaling genes associated with the apoptotic process. Data demonstrated that PGG induced many essential genes that control apoptosis in TNBC cell lines, affecting these genes with different intensities. In MM-231 cells, PGG increased the expression of Caspases 3, 4, and 14 significantly (*p* < 0.01), but in MM-468 cells, only Caspase 3 (*p* < 0.05) was upregulated. An increased expression level for *BIRC3* and *BNIP3* was observed in both cell lines (Fig. 5A). Furthermore, by comparing the mRNA profile of both cell lines, PGG treatment showed an impact on a range of specific genes to one cell line. *BCL2A1*, *BIRC6*, *BNIP3L*, *BLC2L11*, *BNIP2*, *RIPK2*, and *TP53BP2* were upregulated, specifically in MM-231 cells, while *BCL2* and *BAK1* expression was increased only in the MM-468 cells (Fig. 5B,C). Additionally, TNF and TNF receptor superfamily genes were analyzed. In MM-231 cells, the expression of *TNFRSF9*/*CD137* and *TNFRSF10A*/*DR4* was highly induced (11 folds) by PGG treatment, while in MM-468, *TNFRSF10A*/*DR4* presented only a twofold increase, and *TNFRSF9*/*CD137* was down-regulated by 27 folds. *TNFRSF10B*/*DR5* showed a fourfold up-regulation in both cell lines after PGG treatment, and the expression of *TNFRSF21*/*DR6* was only induced in MM-468 cells. Remarkably, PGG increased in 154.6-fold *TNF* expression in MM-468 cells and only 14.6-fold in MM-231 (Fig. 6). The data demonstrates PGG potential in inducing several apoptosis-associated gene expressions, including TNF and TNF receptors in TNBC cells. Data also show how MM-231 and MM-468 breast cancer cells may respond differently to PGG treatment.

## Discussion

Our study's primary focus is to investigate the downstream effects of PGG on the transcription regulation of genes for the treatment of TNBC by comparing genetically different MM-231 and MM-468 cell lines. These cells were previously described as expressing GRO-α/CXCL1 mRNA and protein in significantly increased levels^21^. In the present investigation, the polyphenol PGG downregulated the expression of the chemokine GRO-α/CXCL1 in MM-231 and MM-468 cells stimulated by TNF-α with a higher impact in MM-468 comparing to MM-231 cells, decreasing levels of expression in *GRO-α/CXCL1* mRNA and proteins in these TNBC cells. Reports have described that stromal and immune cells may produce the chemokine CXCL1, which works in a paracrine manner in the tumor environment through cancer development^22,23^. G protein-coupled receptor chemokine (C-X-C motif) receptor 2 (CXCR2) functions as the receptor for CXCL1^24^. The overexpression of CXCL1 has been associated with many malignancy types linked to oncogenesis, metastasis, angiogenesis, and chemoresistance^23,25,26^. In ER-negative cells, higher levels of CXCL1 mRNA and protein expression were detected compared to ER-positive cells. CXCL1 stimulated invasion and migration of BC cells via ERK/MMP2/9 signaling, indicating that tumor-derived CXCL1 may be connected with the high capacity of invasion observed in ER-negative BC cells, thus suggesting that CXCL1 may be a possible target in ER-negative BC therapeutics^27^. In BC, CXCL1-knocked down MM-231 cells presented a decrease of 40% in cell proliferation and even a more significant inhibition in migration (43%) and invasion (60%) studies^21^. Corroborating with the literature, in our studies, the inhibitory effect of PGG over GRO-α/CXCL1 expression may also be associated with the decline in the proliferation rate observed in the MM-231 and MM-468 cell lines since CXCL1 is associated with increased cell growth in MM-231 cells^21^.

To investigate a possible molecular mechanism for PGG inhibitory effect over GRO-α/CXCL1, the levels of expression of genes belonging to NFƙB and MAPK signaling pathways were evaluated after PGG treatment. The results indicate that PGG decreases TNF-α-stimulated GRO-α/CXCL1 release via inhibition of *IқBKE* and MAPK expression. Among the IқB proteins that participate in NFƙB activation, IқBKE showed to be highly expressed in human breast cancer samples, suggesting its participation in the regulation of breast cancer growth, and identifying IқBKE as a new oncogene in breast cancer^28,29^. By silencing *IқBKE* expression in breast cancer cells, studies showed that tumor cell death rate was increased, emphasizing the regulatory role of IқBKE in tumor cell proliferation^28^. Furthermore, another signaling pathway that has a significant role in TNBC progression is the MAPK, which is associated with invasion, metastasis, and prognosis of TNBC^30^. The decision to test MAPK1/ERK2 only in the RT-PCR assays was based on reports that show MAPK3/ERK1 association with a better prognosis and its role in modulating the YAP1 signaling pathway, indicating that MAPK3 is a negative regulator of breast cancer development. However, higher expression of MAPK1 predicted a poor prognosis^31^. The studies in the literature agree with our findings showing that TNF-α stimulates the expression of CXCL1 and supports the fact that PGG inhibitory effect over GRO-α/CXCL1 expression through inhibition of NFƙB and MAPK signaling genes may decrease cell proliferation and slow inflammation and cancer progression.

To further investigate PGG anti-cancer potential, this study also focused on another hallmark of cancer, apoptosis suppression. The investigation of apoptosis-related genes revealed that PGG could stimulate intrinsic and extrinsic apoptotic pathways in MM-231 and MM-468 TNBC cell lines. PGG increased Caspases 3, 4, and 14 expressions in MM-231 cells, but in MM-468, only Caspase 3 was significantly upregulated. Caspase 3 is considered an executioner caspase, and its activation leads to the breakdown of proteins and cytoskeleton cleavage, killing the cell^32^. Caspase 4 and 14 are mainly involved in inflammation and apoptosis. Also, PGG increased mRNA expression of *BNIP3* (BCL2 Interacting Protein 3) and *BIRC3* (Baculoviral IAP repeat-containing 3) in both cell lines, higher expression levels detected in MM-231. BNIP3 role in the cell has been linked to cell death and cell viability^33,34^. *BIRC3* is a gene that codes for inhibitors of apoptosis proteins (IAP) family of proteins cIAP2, which controls caspases and cell death, regulates signaling during inflammation, and controls mitogenic kinase signaling, cell growth, cell invasion, and metastasis^35–37^.

The current results showed variability in PGG ability to induce the two cell lines, anti- and pro-apoptotic genes. In MM-231 cells, PGG induced the expression of *BCL2A1*, *BIRC6*, *BNIP3L*, *BLC2L11*, *BNIP2*, *RIPK2*, and *TP53BP2* genes, while *BCL2* and *BAK1* were increased only in the MM-468 cells. These genes are critical initiators of the intrinsic or extrinsic apoptotic pathways and establish the balance between cell death cell survival. Remarkably, the highest upregulation was seen in tumor necrosis factor (*TNF)* expression with a 154.6-fold increase in MM-468 cells, compared to 14.6-fold in MM-231. Additionally, PGG induced several TNF receptor superfamily members' gene expressions, with MM-231 cells being more responsive to PGG treatment than MM-468. The MM-231 cells presented a higher death receptor 4 (*DR4*), death receptor (*DR5*), and *CD137* expression. In MM-468 cells, a 27-fold upregulation in the death receptor 6 (*DR6*) gene expression was observed, with no significant increase in MM-231 cells. The difference in TNF and TNF receptor expression levels after PGG treatment shows how genetically different TNBC cells may respond differently to a treatment.

Clinical studies with TNF demonstrated that it could be toxic due to its systemic toxicity. However, locoregional drug delivery systems have shown that TNF alone or combined with supplementary pharmacological agents could be a possible approach for tumor therapy by inducing tumor sensitivity to the treatment^38^. The targeted delivery of TNF to the tumor may considerably increase the local concentration in tumor cells, minimizing TNF doses and consequently decreasing the systemic toxicity^38^. Wu et al.^39^ demonstrated that TNF could preserve radiotherapy's sensitizing ability and strength cytotoxicity of chemotherapeutics against breast cancer cells in vitro and in vivo. These results indicate that TNF may be a promising candidate for further clinical application^39^. Therefore, our results show that the PGG effect as an apoptotic inducer through TNF increased expression may have a clinical significance in cancer treatment. Our data also suggests that unique approaches may be considered for different TNBC types since they may have different response levels.

Moreover, members of the TNF superfamily, such as TRAIL, can selectively stimulate apoptosis in numerous tumorigenic cells but not in healthy cells^40^. TRAIL binds to DR4^41^ and DR5^42,43^, which contain functional cytoplasmic death domains that can activate the extrinsic pathway for apoptosis^44^. TRAIL stimulates cell death in a range of cancerous cells despite p53 status. Thus, it may be a helpful strategy in BC therapy, mainly in cells where the response signaling of p53 is not activated, hence avoiding chemotherapy and radiotherapy resistance^45,ε46^. However, TRAIL shows limited therapeutic benefits since most primary cancer cells present resistance to TRAIL. This limitation happens because TRAIL induces apoptosis, but it can also activate cell survival mechanisms, including NFқB, MAPKs, and phosphatidylinositol-3-kinases (PI3K/AKT)^47^. In this regard, the present study showed that PGG upregulated *DR4* and *DR5* mRNA expression in TNBC cells, suggesting that PGG could be combined with TRAIL enhancing the anti-cancer effect of TRAIL. Furthermore, PGG modulatory effect over NFқB signaling, observed in our studies, could also improve apoptosis induced by TRAIL, corroborating with many preclinical models of the tumor where other NFқB inhibitors were used.

**Figure 7:**
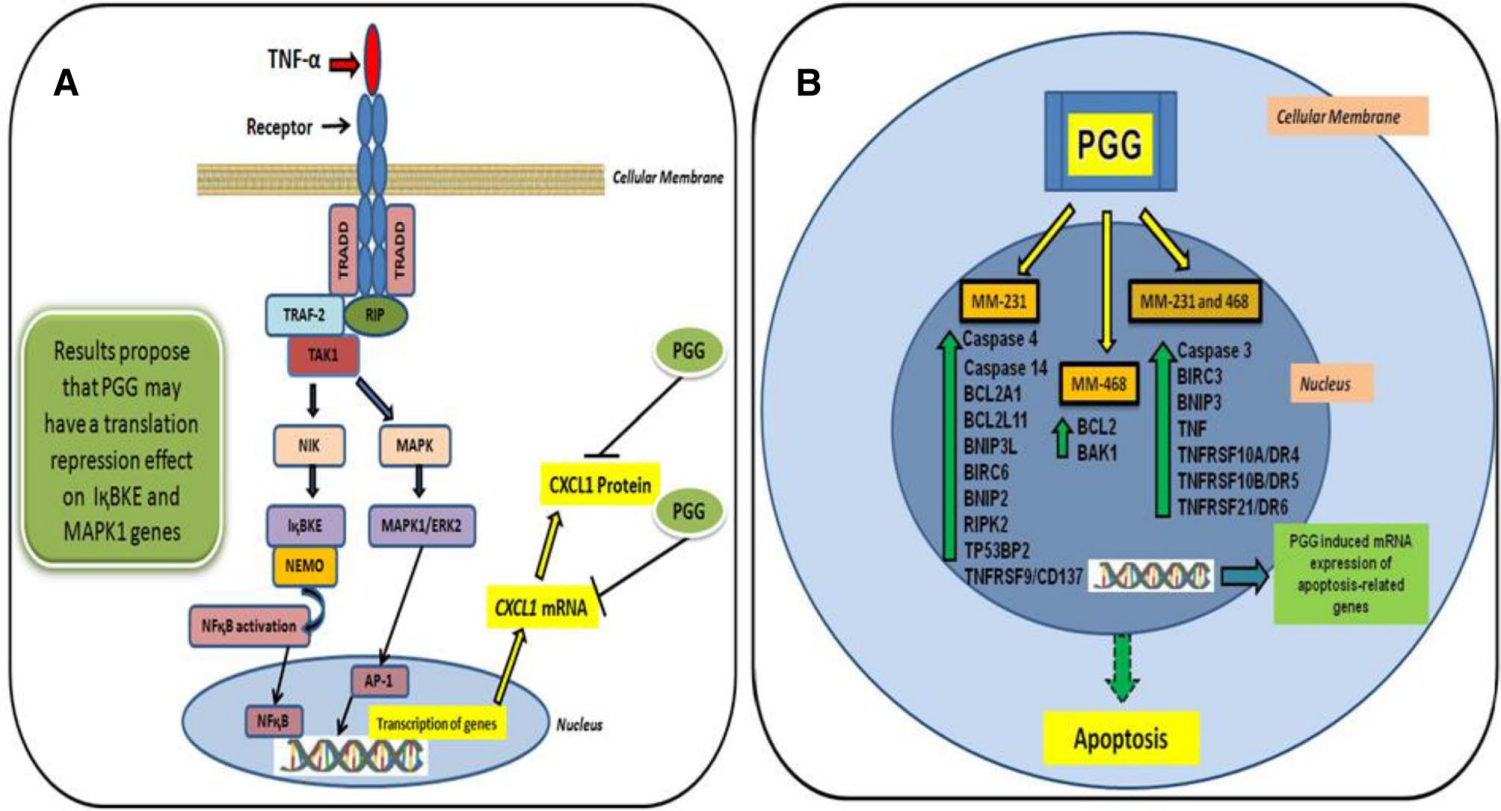
Proposed anti-cancer and apoptotic mechanism of PGG in MM-231 and MM-468 TNBC cells. (**A**) PGG inhibits TNF-α-stimulated CXCL1 mRNA and protein expression, modulating genes involved in the NFқB and MAPK signaling as a possible molecular mechanism. (**B**) PGG-induces expression of specific genes associated with apoptosis in MM-231 and MM-468 TNBC cell lines.

The current study indicates PGG potential as an anti-cancer compound, inhibiting the expression of TNFα-induced CXCL1 via inhibition of the expression of genes and proteins involved in the NFқB and MAPK signaling in MM-231 and MM-468 TNBC cell lines. NFқB was shown to be a crucial modulator of numerous cytokines, including CXCL1. By stimulating the breast cancer cells with TNF-α, we induced a well-known cascade that leads to the phosphorylation of NFқB, which translocates to the nucleus and, together with co-factors, promotes the increased transcription of cytokines^48^. Regarding MAPK signaling, it also has an essential role in activating the transcription of cytokines. By stimulating the cells with TNF-α, MAPK pathway and its associated genes are activated. MAPK/ERK signaling, in particular, is a critical regulator of AP-1 transcriptional activity in TNBC cells^49^. Therefore, Fig. 7 describes the mechanisms used by TNF-α to activate NFқB and MAPK signaling (including activation of NFқB and AP-1 transcription factors) and subsequent transcription of cytokines, such as CXCL1; a mechanism already described in the literature. The figure also shows the proposed mechanism of the PGG effect on IқBKE and MAPK expression, which would lead to a decrease in the expression of CXCL1. PGG inhibitory effect over CXCL1 may also be associated with a cell growth decrease observed in both cell lines.

## Conclusion

The obtained data show that PGG inhibits TNF-α-stimulated CXCL1 mRNA and protein expression and propose that this inhibitory effect may be caused by repression in the translation of genes that modulate NFқB and MAPK signaling as a possible molecular mechanism. The results also suggest PGG potential as an inducer of apoptosis in MM-231 and MM-468 TNBC cells, stimulating expression of numerous apoptosis-associated genes, including caspases. Remarkably, PGG upregulated TRAIL *DR4* and *DR5* receptors mRNA expression and highly induced TNF expression in MM-468 cells. Thus, PGG use may be recommended as an adjunct therapy for TNBC to increase chemotherapy effectiveness and prevent cancer progression. Further investigations are still needed to support the use of PGG in the induction of TNF and its receptors, which might be a helpful strategy to increase tumor sensitivity to chemotherapy.

## Materials and methods

### Cells, chemicals, and reagents

TNBC cells were obtained from American Type Culture Collection (ATCC) (Manassas, VA) and pentagalloyl glucose (purity 96.8%) from Sigma-Aldrich Co. (St. Louis, MO, USA). Human cytokine antibody arrays (Cat# AAH-CYT-6-4), ELISA (Cat# ELH-GRO and Cat# ELH-GRO-alpha), and apoptosis kits (Cat# 68FTAnnV-S100), and tumor necrosis factor-alpha (TNF-α)/human recombinant were obtained from RayBiotech (Norcross, GA, USA). Primers for RT-PCR, SsoAdvanced Universal SYBR (Cat# 1725271), iScript Advanced cDNA Synthesis Kit (Cat# 1725037), apoptosis PCR arrays (Cat# 10034106) were purchased from Bio-Rad (Hercules, CA, USA). Western assays (Cat# SM-W004) were obtained from ProteinSimple (San Jose, CA, USA) and antibodies from Thermo Fisher (Waltham, MA, USA).

### Cell culture and treatments

Dulbecco's modified eagle medium (DMEM) supplemented with 10% fetal bovine serum heat-inactivated (FBS-HI), and 1% penicillin/streptomycin (100 U/ml and 0.1 mg/ml, respectively) were used to grow the MM-231 and MM-468 TNBC cells. DMEM with 2.5% of FBS-HI only was used as experimental media. TNF-α was dissolved in sterile H_2_O, and a working concentration of 50 ng/ml was used in the assays. Dimethyl sulfoxide (DMSO- < 0.1% final concentration) was used to dissolve PGG.

### Cell viability and cell proliferation

Alamar Blue (Resazurin) solution was used to assess PGG's effect on cell viability and cell growth of MM-231 and MM-468 TNBC cell lines. Cells were seeded in 96-well plates (3 × 10^4^ cells/100 μl/well—viability and 5 × 10^3^ cells/100 μl/well—proliferation) overnight and then treated as follows: control (experimental media only), control (cells + DMSO), and cells treated with PGG: 3.125–200 μM for cell viability and 1.56–200 μM for cell proliferation. Treatments were added to each well (100 µl) with 200 μl/well as the final volume. PGG effect on cell viability was detected after 24, 48, and 72 h of incubation. In Proliferation assays, the effect of PGG was measured after 96 h, and 1 µM of taxol and doxorubicin was utilized as positive controls. Alamar Blue in a concentration of 0.5 mg/ml (20 µl) was added to the wells. After 4 h of incubation, the cell viability was determined at an excitation/emission of 550/580 nm wavelengths using a microplate reader Infinite M200 (Tecan Trading AG).

### Human cytokine antibody array membrane

PGG effect on pro-inflammatory cytokines released by TNBC MM-231 and MM-468 cells activated by TNF-α after 24-h treatment was studied using human cytokine antibody arrays. Experiments were carried out in triplicate. Following the protocol, 1 ml of blocking buffer was incubated with the arrays coated with the antibody and then replaced with 1 ml of supernatants of different samples as follows: control (cells + DMSO) samples, PGG-treated cells (6.25 and 25 μM for MM-231 and MM-468 TNBC cells, respectively), TNF-α (50 ng/ml), and the combination of PGG (6.25 or 25 μM) + TNF-α (50 ng/ml). Following overnight incubation at 4 °C, arrays were washed, and 1 ml of biotin-conjugated antibodies was added. After 2 h-incubation, HRP-conjugated streptavidin was added and left for 2 h. Lastly, with the addition of a chemiluminescent reagent, the image of spots were taken (Flour-S Max multi-imager- Bio-Rad Laboratories, Hercules, CA, USA), and the density of spots determined (Quantity One Software- Bio-Rad Laboratories, Hercules, CA).

### Human GRO and GRO-α ELISA assay

PGG effect on GRO and GRO-α expression was investigated using individual quantitative ELISA assays. Supernatants from the control (cells + DMSO), PGG-treated (6.25- MM-231 and 25 µM- MM-468), TNF-αstimulated (50 ng/ml), and co-treated (PGG + TNF-α) cells were collected, and the cells centrifuged at 1000 rpm for 4 min. Experiments were performed in triplicate according. Supernatants from samples and standards (100 μl) were pipetted into the 96 well plates and incubated at room temperature. After 2.5 h, the plates were washed, and the biotinylated antibody was added. Following 1 h, streptavidin (100 μl) was added, and within 45 min of incubation, the substrate reagent was incubated for 30 min. The quantified data was determined by optical density at 450 nm (Synergy HTX multi-Reader- BioTek, USA).

### Real time-polymerase chain reaction (RT-PCR)

The pellet of cells for the RT-PCR reaction was obtained after 24 h of the following treatments: control (cells + DMSO), PGG-treated (6.25 µM for MM-231 and 25 µM for MM-468 cells), TNF-α-stimulated (50 ng/ml) and co-treated with PGG + TNF-α. RNA was extracted using the Trizol reagent. iScript advanced reverse transcriptase was used to synthesize cDNA strands from the mRNA. The advanced reaction mix, reverse transcriptase, sample (1.5 µg/reaction), and water were combined in a volume of 20 µl. The reverse transcription protocol for thermal cycling consisted of two steps: 46 °C for 20 min and 95 °C for 1 min. For the RT-PCR assay, the sample (200 ng cDNA/reaction), master mix, primer, and water were placed together in each well. According to the protocol from Bio-Rad, the program for the thermal cycling consisted of an initial step at 95 °C for 2 min and denaturation at 95 °C for 10 s, followed by 39 cycles of 60 °C for 30 s (annealing/extension), and 65–95 °C for 5 s/step (melting curve) (Bio-Rad CFX96 Real-Time System- Hercules, CA, USA). The UniqueAssay ID for the primers is described as follows:

*GRO-α*: qHsaCED0046130; IқBKE: qHsaCID0014831; *MAPK1*: qHsaCED0042738.

### Capillary electrophoresis Western analysis

Cell pellets corresponded to control (cells + DMSO), PGG-treated (6.25 µM- MM-231 and 25 µM- MM-468 cells), TNF-α-stimulated (50 ng/ml) and co-treated (PGG + TNF-α) cells after 24-h treatment. A lysis buffer containing a protease inhibitor cocktail was added to each pellet, and total protein expression was determined using Western analysis. Samples containing 0.2 mg/ml of protein were used. Following the manufacture's protocol (ProteinSimple), the microplate was loaded and placed in the instrument. The reaction took place inside the capillary system; using specific antibodies, the proteins were identified, the chemiluminescence reaction was determined, and the digital blot images were taken. The antibodies used in this assay are:

$$1)\;{\text{p}}44/42\;{\text{MAPK}}\;\left( {{\text{Erk}}1/2} \right)\;\left( {137{\text{F}}5} \right)\;{\text{Rabbit}}\;{\text{mAb}}\# 4695\;{\text{and}}\;2)\;{\text{IKK}}\upvarepsilon \;{\text{Antibody}}\;\# 2690$$

### Apoptosis detection using annexin V-FITC and apoptosis PCR arrays

Apoptosis Kit (Annexin V-FITC) was used to establish PGG apoptotic effect on MM-231 and MM-468 TNBC cells. The cells were seeded (5 × 10^5^ cell/well) and incubated overnight using 6-well plates. The next day cells were treated with PGG in a concentration ranging from 25 to 200 µM (final volume 3 ml/well). Control cells consisted of cells + DMSO (concentration < 0.1%). Following 24 h of incubation, cell pellets were collected and resuspended in 500 µl of the buffer. Following the manufacture's protocol (RayBiotech), 5 µl of Annexin V-FITC and 5 µl propidium iodide was added to the samples. PGG apoptotic effect was determined by flow cytometry (FACSCalibur- Becton Dickinson, San Jose, CA, USA), and data analysis was performed with CellQuest software. For the apoptosis PCR arrays, cell pellets from control (cells + DMSO) and PGG-treated cells (100 µM for MM-231 and 50 µM for MM-468 cells) were lysed with Trizol reagent. iScript advanced reverse transcriptase was used to synthesize cDNA strands from the mRNA, as described previously. For the RT-PCR reaction, the sample (10 ng cDNA/reaction) and master mix were combined in the plate. The thermal cycling program was the same one used previously.

### Data analysis

GraphPad Prism (version 6.07) (San Diego, CA, USA) was used to determine the results' statistical significance. All the experiments were performed at least in triplicates. The IC_50_ calculation in cell viability studies was calculated using nonlinear regression. The statistical significance assessment between the treatments was established using a one-way ANOVA analysis and Dunnett's multiple comparison tests. Human Cytokine Array software C1000 (Code: S02-AAH-CYT-1000- RayBiotech) was used to establish cytokine expression on the arrays. The expression of specific genes was determined with CFX 3.1 Manager software (Bio-Rad, Hercules, CA). Total protein expression was determined using ProteinSimple Compass software, as well as the digital blot images.

### Consent for publication

The authors agree for publication.

## Supplementary Information

Supplementary Information

## Data Availability

All relevant data are within the manuscript.

## Abbreviations

ATCC: American type culture collection
BC: Breast cancer
BIRC3: Baculoviral IAP repeat containing 3
BNIP3: BCL2 interacting protein 3
CXCL1: C-X-C motif ligand 1
CXCR2: C-X-C motif receptor 2
DMEM: Dulbecco's modified eagle medium
DMSO: Dimethyl sulfoxide
DR: Death receptor
ER: Estrogen receptor
FBS-HI: Fetal bovine serum heat-inactivated
GRO-α: Growth-regulated oncogene-alpha
MM-231: MDA-MB-231
MM-468: MDA-MB-468
PGG: Pentagalloyl glucose
PI3K/AKT: Phosphatidylinositol-3-kinases
TME: Tumor microenvironment
TNBC: Triple-negative breast cancer
TNF: Tumor necrosis factor
TRAIL: TNF-related apoptosis-induced ligand
