# Supplementary material for: Pentagalloyl glucose inhibits TNF‐α‐activated CXCL1/GRO-α expression and induces apoptosis‐related genes in triple-negative breast cancer cells
Source: Sci Rep. 2021 Mar 11;11:5649. doi: 10.1038/s41598-021-85090-z (PMC7952910; doi:10.1038/s41598-021-85090-z)
Supplement: Supplementary file 1 — Supplementary Information [file 41598_2021_85090_MOESM1_ESM.docx]

***Supplemental Materials to:***

**Pentagalloyl glucose inhibits TNF**‐**α**‐**activated CXCL1/GRO-α expression and induces apoptosis**‐**related genes in triple-negative breast cancer cells.**

**Patricia Mendonca, Sumaih Alghamdi, Samia Messeha and Karam F.A. Soliman***

**Division of Pharmaceutical Sciences, College of Pharmacy and Pharmaceutical Sciences, Institute of Public Health, Florida A&M University, Tallahassee, FL 32307, USA.**

*** Corresponding author:**

Karam F.A. Soliman, PhD

Division of Pharmaceutical Sciences,

College of Pharmacy and Pharmaceutical Sciences, Institute of Public Health Florida A&M University

Room G134 H Pharmacy Building

1415 ML King Blvd

Tallahassee, FL 32307, USA

Tel 850 599 3306 Fax 850 599 3667

Email: karam.soliman@famu.edu

***Original Blots Cropped from Compass Software (ProteinSimple Company)***

**Bands correspond to the following treatments:**

**Control, PGG, TNF-α, and PGG + TNF-α, respectively.**

**____________________________________________________________________________**

**MM-231 - Protein: IKBKE**


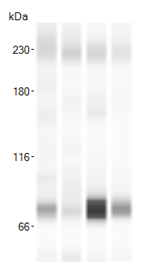
**_____________________________________________________________________________________**

**IKBKE**

**MM-231 - Protein: MAPK1**


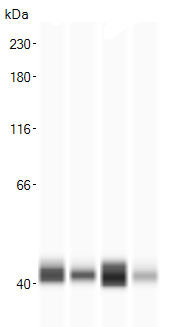
**_____________________________________________________________________________________**

**MAPK1**

**____________________________________________________________________________**

**MM-468 - Protein: IKBKE**

**
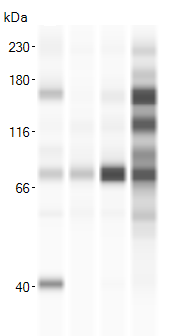
_____________________________________________________________________________________**

**IKBKE**

**MM- 468 - Protein: MAPK1**

**
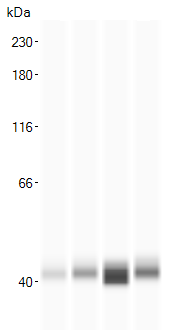
_____________________________________________________________________________________**

**MAPK1**

**_____________________________________________________________________________________**

**MM-231 - Protein GAPDH**

**
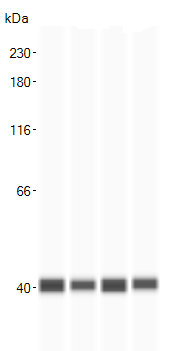
_____________________________________________________________________________________**

**GAPDH**

**MM-468 - Protein GAPDH**

**
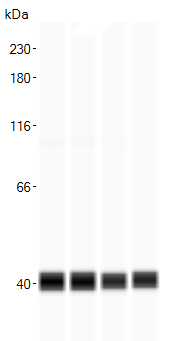
_____________________________________________________________________________________**

**GAPDH**
